# Genes and Aggressive Behavior: Epigenetic Mechanisms Underlying Individual Susceptibility to Aversive Environments

**DOI:** 10.3389/fnbeh.2018.00117

**Published:** 2018-06-13

**Authors:** Sara Palumbo, Veronica Mariotti, Caterina Iofrida, Silvia Pellegrini

**Affiliations:** ^1^Department of Surgical, Medical, Molecular Pathology and Critical Care, University of Pisa, Pisa, Italy; ^2^Department of Experimental and Clinical Medicine, University of Pisa, Pisa, Italy; ^3^Department of Pharmacy, University of Pisa, Pisa, Italy

**Keywords:** epigenetics, aversive environment, aggressive behavior, NR3C1, OXTR, SLC6A4, MAOA

## Abstract

Over the last two decades, the study of the relationship between *nature* and *nurture* in shaping human behavior has encountered a renewed interest. Behavioral genetics showed that distinct polymorphisms of genes that code for proteins that control neurotransmitter metabolic and synaptic function are associated with individual vulnerability to aversive experiences, such as stressful and traumatic life events, and may result in an increased risk of developing psychopathologies associated with violence. On the other hand, recent studies indicate that experiencing aversive events modulates gene expression by introducing stable changes to DNA without modifying its sequence, a mechanism known as “epigenetics”. For example, experiencing adversities during periods of maximal sensitivity to the environment, such as prenatal life, infancy and early adolescence, may introduce lasting epigenetic marks in genes that affect maturational processes in brain, thus favoring the emergence of dysfunctional behaviors, including exaggerate aggression in adulthood. The present review discusses data from recent research, both in humans and animals, concerning the epigenetic regulation of four genes belonging to the neuroendocrine, serotonergic and oxytocinergic pathways—Nuclear receptor subfamily 3-group C-member 1 (*NR3C1*), oxytocin receptor (*OXTR*), solute carrier-family 6 member 4 (*SLC6A4*) and monoamine oxidase A (*MAOA*)—and their role in modulating vulnerability to proactive and reactive aggressive behavior. Behavioral genetics and epigenetics are shedding a new light on the fine interaction between genes and environment, by providing a novel tool to understand the molecular events that underlie aggression. Overall, the findings from these studies carry important implications not only for neuroscience, but also for social sciences, including ethics, philosophy and law.

## New Frontiers in Epigenetic Research

Studies both in animals (Mosaferi et al., 2015) and humans (McEwen et al., 2012) indicate that the environment, mostly during prenatal stage and infancy, impact significantly on neural development, as several critical periods with lasting consequences on behavior have been documented (Stiles and Jernigan, 2010). Moreover, rodent studies have found that adolescent expression of 5-HT1B receptors has a direct impact on later patterns of aggressive behavior (Nautiyal et al., 2015). Therefore, the flexibility of neural programing during critical periods seems to be a significant mediator of long-lasting effects on behavior (Morrone, 2010). In particular, adversities experienced during prenatal life and infancy interfere with the normal processes of cell proliferation and differentiation leading to altered neural circuits that may result in cognitive and emotional deficits. These alterations have been associated with both proactive (e.g., children callous-unemotional traits) and reactive aggressive behavior (e.g., children externalizing disorder spectrum) that may anticipate Antisocial Personality Disorder (Frick and White, 2008; Buchmann et al., 2014; Mann et al., 2015; Rosell and Siever, 2015).

Aggression, throughout evolution, serves an important role in the survival of a species (Darwin, 1859, 1871). Being aggressive gives the best chances for survival and reproduction (Veroude et al., 2016). This is true for all mammalian species, including human. However, when excessive, the consequences of aggressive acts can be maladaptive (Takahashi and Miczek, 2014; Waltes et al., 2016).

Experiencing repeated aversive life events or protracted stress during pregnancy, especially during the first trimester of gestation, results in increased risk of physically-aggressive tendencies, delinquency and conduct disorder, both in early childhood and adolescence (Kvalevaag et al., 2014; Van den Bergh et al., 2017). During the first trimester, the neuroectoderm develops and becomes the source of neural progenitor cells, as well as the foundation of the neural tube (Stiles and Jernigan, 2010). Similar outcomes are predictable by postnatal traumas. The risk of aggressive behavior in childhood is particularly high in infants neglected during their first 2 years of life, when the brain doubles its volume and a massive synaptogenesis occurs (Knickmeyer et al., 2008; Tau and Peterson, 2010). Neglecting to provide early-life basic physical needs and emotional support as a parent can later lead to higher scores of aggression in childhood, measured by the Child Behavior Checklist (Kotch et al., 2008). Moreover, recurrent experiences of emotional abuse or witnessing violence throughout childhood predict physical aggressive behavior in adulthood (Sansone et al., 2012).

Studies in animals support the implication of prenatal and childhood adversities in the origin of aggressive behavior. In juvenile and adult male rats, for example, an increased number of physical attacks toward inoffensive peers and females have been predicted by repeated maternal separation in the first 2 weeks of life or by post-weaning social isolation (Haller et al., 2014). In rats, both prenatal and early postnatal stressors, like physical restraint during pregnancy or repeated maternal separation after birth, interfere with normal cell proliferation and differentiation and with dendritic formation, leading to altered neural circuits that may result in exaggerate aggressive behaviors (Lukas et al., 2010; de Souza et al., 2013). These early aversive experiences affect the functioning of many biochemical pathways (e.g., vasopressin, oxytocin, serotonin and cortisol pathways) that play a crucial role for the development of social skills and for the response to stress; the persistence of these alterations predisposes juvenile rats to excessive offensive play-fighting and then, as adults, to high levels of offensive attacks toward peers (Veenema et al., 2006; Veenema and Neumann, 2009; Veenema, 2009; Lukas et al., 2010; de Souza et al., 2013; Haller et al., 2014).

In addition to prenatal and early postnatal life, adolescence also represents a time-window particularly sensitive to external/environmental events, as in this period the brain concludes its maturation process (Morrone, 2010). As demonstrated by rats, during this period of life, a massive reorganization occurs in specific brain areas—hippocampus, cortex and amygdala—whose morphological and functional alterations have been linked to violence in humans (Isgor et al., 2004; Morrison et al., 2014); an increased amygdala volume, for example, has been observed in incarcerated criminals (Schiffer et al., 2011). Furthermore, peripubertal exposure of rats to fear-inducing stressors, such as the presence or the smell of a predator, predicts the expression of aggressive behavior later in adulthood (Cordero et al., 2013; Márquez et al., 2013).

It has been known for some time that genetic variants, which regulate aminergic signaling in brain, modulate vulnerability to aversive environmental factors resulting in different behavioral phenotypes (for a review see Iofrida et al., 2014; Veroude et al., 2016). More recently, it has emerged that environmental factors stably affect gene expression by producing specific signals to DNA, chromatin and mRNA that do not modify the nucleotide sequence (Bale, 2015). This phenomenon, known as epigenetics, probably mediates the long-lasting effects of aversive experiences on brain and behavior, through the generation of new trajectories of neuronal development (Morrison et al., 2014; Bale, 2015).

## The Epigenetic Mechanisms: An Overview

The main epigenetic changes playing an active role in gene expression regulation are represented by DNA methylation, post-translational histone modifications and post-transcriptional regulation by microRNAs (miRNAs; Dolinoy et al., 2007; Chhabra, 2015).

DNA methylation is carried out by three active isoforms of the DNA methyltransferase family (DNMT-1, -3a and -3b), which are ubiquitous nuclear enzymes, able to transfer residues of methyl groups from the S-adenosylmethionine (SAM) to unmethylated cytosines, preferably cytosine-guanine dinucleotides (CpGs; Chiang et al., 1996). Most CpGs are grouped in specific loci of the genome, the CpG islands, which are located into promoters, exons and, to a lower extent, introns (Schwartz et al., 2009; Gelfman et al., 2013). DNMTs inhibit DNA transcription by blocking the interactions among DNA, RNA polymerase II and transcription factors, by promoting the heterochromatin formation and by interfering with the splicing process (Maunakea et al., 2013). DNMT-1, also called maintenance methyltransferase, methylates the newly replicated strand of DNA by copying the methylation patterns from the parent strand. Its role is to preserve the correct DNA methylation pattern during mitosis in daughter cells (Bird, 2002). DNMT-3a and -3b perform *de novo* methylation of unmethylated CpGs and produce new DNA methylation marks. The *de novo* methylation mainly occurs in the early embryonic cells and, not surprisingly, both enzymes are highly expressed in these cells (Okano et al., 1999).

Post-translational histone modifications are covalent modifications of the amino-terminal tails of the histones including acetylation, phosphorylation, methylation and ubiquitylation. Such modifications influence the interaction between DNA and histones, thus modifying the chromatin compacting state (Bannister and Kouzarides, 2011). Histone acetylation is mediated by the histone acetyltransferase (HAT) enzymes that cause chromatin decondensation by transferring acetyl groups from the acetyl-Coenzyme A to lysine residues within the amino-terminal tails of nucleosomal histones. The addition of acetyl groups neutralizes the positive charge of lysines, weakening the interaction between histones and DNA, thus making DNA accessible to the transcriptional machinery (Bannister and Kouzarides, 2011). At the opposite, the histone deacetylases (HDACs) remove acetyl groups from lysine residues to restore their positive charge. Histone deacetylation allows histones to tightly bind DNA, thus favoring a more compact configuration of chromatin and a consequent inhibition of transcription (Lombardi et al., 2011). Histone phosphorylation predominantly occurs on threonine, tyrosine and serine residues and is mediated by kinases that transfer a phosphate group from ATP to the hydroxyl group of the target amino-acid side chain. The addition of phosphate groups negatively charges histones, thus weakening their interaction with DNA. The histone dephosphorylation is catalyzed by phosphatases (Bannister and Kouzarides, 2011). Histone methylation takes place on the side chains of lysines and arginines, within the histone tails (Kouzarides, 2007). Histone Lysine Methyltransferase (HKMT) and Protein Arginine Methyltransferase (PRMT) are the enzymes that catalyze the transfer of a methyl group from SAM to lysine and arginine residues, respectively (Bannister and Kouzarides, 2011). Both lysine and arginine methylations act either as activators or as repressors for transcription (Kouzarides, 2007). Lysine residues are de-methylated by both the lysine-specific demethylase 1 (LSD1) and the jumonji domain 2 protein (JMJD2), whereas the jumonji domain 6 protein (JMJD6) de-methylates the arginine residues (Bannister and Kouzarides, 2011). Histone ubiquitylation consists of a binding between histone lysine residues and ubiquitin, through the sequential action of three enzymes: E1-activating, E2-conjugating and E3-ligating enzymes. Also this histone modification can be either activatory or repressive for transcription. Ubiquitin is removed by specific isopeptidases named de-ubiquitin enzymes (Bannister and Kouzarides, 2011).

miRNAs are untranslated transcripts that originate from MIR genes, located in clusters within the introns of other genes (Bhat et al., 2016). MIR genes are transcribed by RNApol II or III in long primary transcripts called pri-miRNAs that undergo extensive processing to generate mature double-stranded miRNAs. One strand is complementary to the 3’untraslated region of the target mRNA, where it binds, thus blocking gene expression either temporarily, through the mRNA translational repression, or permanently, through the mRNA cleavage (Issler and Chen, 2015).

The above-described chromatin modifications are extremely dynamic and subjected to continuous changes in response to external stimuli. As such, these molecular processes are vulnerable to modifications before and after childbirth, rendering gene expression plastic throughout mammalian life. Thus, they represent promising targets for behavioral treatment strategies. Talking about aggressive behavior predisposition, however, a role has been described to date only for DNA methylation and histone acetylation, whose scientific evidence in literature is reviewed below.

## Genes Whose Epigenetic Marks Are Involved in Human Aggressive Behavior

### Nuclear Receptor Subfamily 3-Group C-Member 1 (Glucocorticoid Receptor; NR3C1)

Nuclear receptor subfamily 3-group C-member 1 (*NR3C1*) encodes for a nuclear glucocorticoid receptor that interacts with cortisol to control the functioning of the hypothalamic-pituitary-adrenocortical (HPA) axis *via* a negative feedback that ultimately inhibits cortisol release (Kino and Chrousos, 2002).

According to a meta-analysis published in 2009 (Hawes et al., 2009), a great amount of data shows that cortisol is reduced in antisocial behavior. Low basal levels of blood cortisol, for example, have been associated with externalizing behavior in childhood (Alink et al., 2008) and adolescence (Shoal et al., 2003; Shirtcliff et al., 2005). In adolescence, low plasma concentration of cortisol has been negatively correlated also to low self-control (Shoal et al., 2003), delinquent behavior and proactive and reactive aggression (Poustka et al., 2010). Interestingly, a history of child abuse and neglect predicted lower HPA activity and higher trait and state aggression in adults, suggesting that the HPA hypo-activity may be a mediator between environment and long-lasting aggressive behavior (Gowin et al., 2013). A recent study confirmed this hypothesis; specifically, the hypo-methylation of *NR3C1*, which translates into augmented inhibitory control of HPA axis, has been shown to be induced by early adverse family environment, and to represent a risk factor for aggressive externalizing behavior in adolescence (Heinrich et al., 2015). As these epigenetic changes are produced during infancy when brain development is maximal, they persist well beyond in life (Radtke et al., 2011). They significantly impact neurodevelopment and predispose to behavioral alterations, including impaired stress response and poor self-regulation (Conradt et al., 2013), which concur in predisposing to aggressive behavior.

### Oxytocin Receptor (OXTR)

Oxytocin is a hypothalamic hormone, also known as the “social neuropeptide”, that regulates complex social behaviors by promoting attachment and facilitating social interactions (Meyer-Lindenberg et al., 2011). Impaired functioning of the oxytocinergic system has been observed in rodents with aggressive behavior (Lubin et al., 2003; McMurray et al., 2008), and a lower oxytocin concentration in the central nervous system represents a predisposing factor to human aggressive behavior (Lee et al., 2009; Jokinen et al., 2012).

Social environment induces changes in the oxytocinergic system, especially during the early postnatal period and the infancy (Veenema, 2012). Oxytocin secretion (measured in saliva and whole blood), for instance, is stimulated in infants and children by maternal care (Wismer Fries et al., 2005; Tsuji et al., 2015), while childhood maltreatments, especially emotional abuses, result in lower levels of oxytocin in the cerebral spinal fluid of adults (Heim et al., 2009). Similarly, a lower expression of the oxytocin receptor (OXTR) has been detected in rodents and macaques poorly nurtured (Francis et al., 2000; Baker et al., 2017).

DNA methylation of *OXTR* is an important mechanism linking aversive experiences to susceptibility to abnormal behavior in adulthood (Veenema, 2012; Unternaehrer et al., 2015; Ziegler et al., 2016). A history of repeated early abuses and traumatic experiences, in fact, has been correlated to increased *OXTR* methylation in depressed and anxious adults (Smearman et al., 2016; Gowin et al., 2017).

*OXTR* methylation is affected by negative events also before birth. In particular, newborns from women who suffered from drug addiction, psychopathy or showed criminal behaviors during pregnancy, carried hyper-methylated *OXTR* and had an increased probability of developing callous-unemotional traits (Cecil et al., 2014), indicative of stable and severe aggressive behavior (Frick and White, 2008).

### Serotonin Pathway

Serotonin plays a key role in most of psychiatric conditions and in antisocial/aggressive personality (Nutt, 2008; Seo et al., 2008). Brain serotonin concentration is regulated by serotonin transporter solute carrier-family 6 member 4 (SLC6A4) that controls its reuptake from the synaptic cleft, and by monoamine oxidase A (MAOA) that catabolizes serotonin (Shih et al., 1999).

Hypo-functioning of serotonin neurotransmission has been linked to higher risk of aggressive behaviors (Davidson et al., 2000). For instance, the brain expression of SLC6A4 is reduced in aberrant impulsive-aggressive individuals (Frankle et al., 2005). Consistently, early aversive experiences exert epigenetic regulation of *SLC6A4* with implications in the development of such conditions (Provencal and Binder, 2015). Childhood stress, e.g., bullying victimization by peers, increased the saliva methylation of *SLC6A4* promoter from age 5 to age 10 (Ouellet-Morin et al., 2013). Moreover, as observed in females, being physically (including sexually) abused by parents from childhood to adolescence predicts, in adulthood, both an increased *SLC6A4* methylation in peripheral white cells (Beach et al., 2010) and a higher risk of developing long-lasting antisocial personality disorders (Beach et al., 2011, 2013). An *in vivo* study in males found a similar link between physical abuses experienced in childhood and *SLC6A4* hyper-methylation in peripheral lymphocytes correlating with low brain (orbitofrontal cortex) synthesis of serotonin (Wang et al., 2012). These data suggest that *SLC6A4* is silenced by early stressors as a protective mechanism aimed at the potentiation of the serotonergic neurotransmission; however, a long-lasting hyper-methylation results in lower cortical thickness (Park et al., 2015; Won et al., 2016) and alters amygdala reactivity (Nikolova et al., 2014), thus probably predisposing to aggressive behavior. For example, adolescents that have been raised in low socioeconomic status show higher methylation of *SLC6A4* in peripheral lymphocytes and higher amygdala activation in response to fearful faces (Swartz et al., 2017). As far as the orbitofrontal cortex concerns, an increased activity of this brain area predicted aggressive responses to angry faces (Beyer et al., 2015); moreover, morphological asymmetry of this area has been associated with higher scores at the Lifetime History of Aggression, and Buss-Perry Aggression scales (Antonucci et al., 2006).

Finally, in a rat model of pathological aggression, the exposure to peripubertal stress affected the connectivity between amygdala and orbitofrontal cortex accompanied by a parallel increase of *MAOA* expression in the frontal cortex in adulthood. Interestingly, an increased H3 acetylation of *MAOA* was observed in the prefrontal cortex suggesting that the aversive experience has induced a stable epigenetic regulation of the transcription of this gene (Márquez et al., 2013).

## Conclusion

In recent years, neuroscientific research has focused more and more on the biological mechanisms that predispose to behavioral disorders as a consequence of the exposure to aversive environments. Specific genetic variants, in interaction with negative environmental experiences during prenatal life, childhood and adolescence, have been shown to affect the development of long-lasting aggressive behavior and psychiatric disorders in adulthood, with significant social, legal and moral implications (Rigoni et al., 2010; Sartori et al., 2011; Jones et al., 2013; Roth, 2013; Iofrida et al., 2014; Rota et al., 2016; Pellegrini et al., 2017). As a matter of fact, recent studies suggest that the same genetic variants that increase the risk of aggressive behavior in combination with a negative environment, may actually act as plasticity variants, making the brain more sensitive also to positive environmental inputs, resulting in increased prosocial behavior (Belsky et al., 2009; Simons et al., 2011; Iofrida et al., 2014).

Aggression actually represents an evolutionary important behavior fostered by stressful life events, fundamental to deal with life threating situations and to preserve one’s own life (Stiles and Jernigan, 2010). However, if exaggerate and uncontrolled, it represents a pathological condition characterizing externalizing behavior, conduct disorders, callous-unemotional traits and psychopathy (Beach et al., 2011; Kumsta et al., 2013; Cecil et al., 2014; Heinrich et al., 2015; Kundakovic et al., 2015).

Over the last few years, the epigenetic mechanisms underlying human aggressive behavior have been attracting a growing interest, as they provide a fascinating and reliable explanation of the gene-environment interplay that modulates human violent behavior. Epigenetics, indeed, plays a central role in the adaptation of the human organism to the changing environment. This concept emerged first from studies conducted in monozygotic twins, which showed that different phenotypes may originate from identical genotypes due to epigenetic changes (Poulsen et al., 2007). These differences progressively increase as twins become older, along with the diversification of their lifestyles and living environments (Fraga et al., 2005).

**Figure 1:**
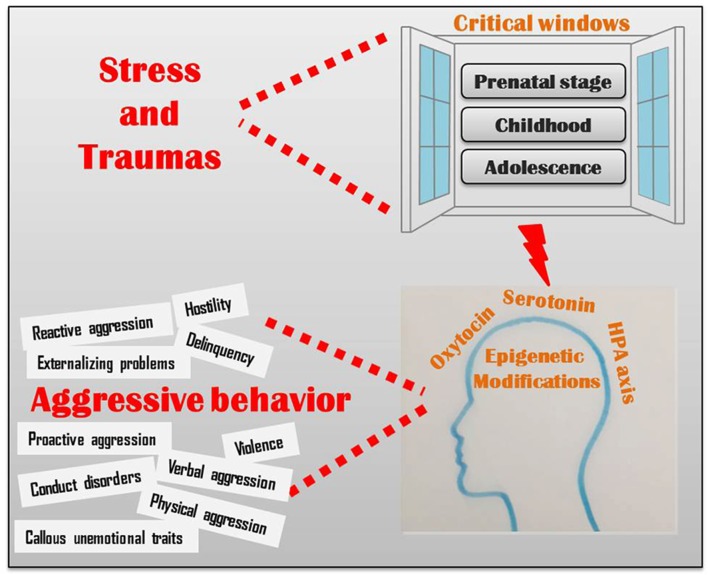
Critical windows of environmental vulnerability to aggressive behavior.

Although the existence of a genetic blueprint underlying brain development is undeniable, the epigenetic control of biological pathways, including the neuroendocrine, serotonergic and oxytocinergic pathways, significantly mediates the behavioral responses to the environment (Figure 1; Veenema, 2012; Waltes et al., 2016). Epigenetic changes in these pathways may alter brain morphology and functioning in areas that hold a crucial role in cognitive and emotional processes underlying aggression (Conradt et al., 2013; Ouellet-Morin et al., 2013; Suri et al., 2013; Booij et al., 2015; Puglia et al., 2015; Gowin et al., 2017). Recent data indicate that these epigenetic marks may be, in some extent, reversed by the exposure to an enriched environment therapy; for example, massage therapy significantly reduced aggressive behavior in children and adolescence (Diego et al., 2002; Garner et al., 2008), probably by epigenetic mechanisms (McCreary and Metz, 2016). Alternatively, it is possible to intervene by a pharmacological therapy, as shown in rats: treating aggressive adult rats that had experienced peripubertal stress with a MAOA inhibitor reversed their aberrant behavior (Márquez et al., 2013).

In conclusion, epigenetics is shedding a new light on the fine interaction between *nature* and *nurture*, by providing a novel tool to understand the molecular events that underlie the relationship among genes, brain, environment and behavior. Altogether, the results of the studies that we briefly discussed in the present article, clearly indicate that, when it comes to (human) behavior, *nature* and *nurture* are not to be regarded as two distinct and separate factors, contrary to the alternating predominance of either one that has been proposed in different historic phases (Levitt, 2013; Moore, 2016). Indeed, distinct genetic backgrounds differentially modulate the individual susceptibility to the environment and at the same time various environmental conditions differentially affect gene expression, in an intimate and fascinating manner that scientists have now begun to disentangle. The findings from this research pave the way to a novel approach to the understanding of human behavior, with important implications also for social sciences, including philosophy, ethics and law. Unveiling the molecular mechanisms that regulate the expression of human behavior will provide a solid scientific basis to what philosophy already sensed since its dawn, suffice it to mention what the great Plato wrote over 25 centuries ago: “*No one is willingly evil, but one can become evil for a bad disposition in his body and for a training without a true education; this is hideous for everyone and happens against his will”* (Timeus, 86e).

## Author Contributions

SPalumbo, VM and CI searched and reviewed the scientific literature; all the authors discussed the findings from the literature; SPalumbo and VM drafted the manuscript; SPellegrini conceived the work and revised the manuscript.

## Conflict of Interest Statement

The authors declare that the research was conducted in the absence of any commercial or financial relationships that could be construed as a potential conflict of interest.
